# A New Antibiotic-Loaded Sol-Gel Can Prevent Bacterial Prosthetic Joint Infection: From *in vitro* Studies to an *in vivo* Model

**DOI:** 10.3389/fmicb.2019.02935

**Published:** 2020-01-17

**Authors:** John Jairo Aguilera-Correa, Amaya Garcia-Casas, Aranzazu Mediero, David Romera, Francisca Mulero, Irene Cuevas-López, Antonia Jiménez-Morales, Jaime Esteban

**Affiliations:** ^1^Clinical Microbiology Department, IIS-Fundacion Jimenez Diaz, UAM, Madrid, Spain; ^2^Department of Materials Science and Engineering, University Carlos III of Madrid, Madrid, Spain; ^3^Bone and Joint Research Unit, IIS-Fundacion Jimenez Diaz, UAM, Madrid, Spain; ^4^Molecular Imaging Unit, Spanish National Cancer Research Centre (CNIO), Madrid, Spain; ^5^Experimental Surgery and Animal Research Service, IIS-Fundacion Jimenez Diaz, UAM, Madrid, Spain; ^6^Álvaro Alonso Barba Technological Institute of Chemistry and Materials, Carlos III University of Madrid, Madrid, Spain

**Keywords:** sol-gel, moxifloxacin, prosthetic joint infection, *Staphylococcus epidermidis*, *Staphylococcus aureus*, *Escherichia coli*

## Abstract

The aim of this study was to evaluate the effect of a moxifloxacin-loaded organic-inorganic sol-gel with different antibiotic concentration in the *in vitro* biofilm development and treatment against *Staphylococcus aureus*, *S. epidermidis*, and *Escherichia coli*, cytotoxicity and cell proliferation of MC3T3-E1 osteoblasts; and its efficacy in preventing the prosthetic joint infection (PJI) caused by clinical strains of *S. aureus* and *E. coli* using an *in vivo* murine model. Three bacterial strains, *S. epidermidis* ATCC 35984, *S. aureus* 15981, and, *E. coli* ATCC 25922, were used for microbiological studies. Biofilm formation was induced using tryptic-soy supplemented with glucose for 24 h, and then, adhered and planktonic bacteria were estimated using drop plate method and absorbance, respectively. A 24-h-mature biofilm of each species growth in a 96-well plate was treated for 24 h using a MBECTM biofilm Incubator lid with pegs coated with the different types of sol-gel, after incubation, biofilm viability was estimated using alamrBlue. MC3T3-E1 cellular cytotoxicity and proliferation were evaluated using CytoTox 96 Non-Radioactive Cytotoxicity Assay and alamarBlue, respectively. The microbiological studies showed that sol-gel coatings inhibited the biofilm development and treated to a mature biofilm of three evaluated bacterial species. The cell studies showed that the sol-gel both with and without moxifloxacin were non-cytotoxic and that cell proliferation was inversely proportional to the antibiotic concentration containing by sol-gel. In the *in vivo* study, mice weight increased over time, except in the *E. coli*-infected group without coating. The most frequent symptoms associated with infection were limping and piloerection; these symptoms were more frequent in infected groups with non-coated implants than infected groups with coated implants. The response of moxifloxacin-loaded sol-gel to infection was either total or completely absent. No differences in bone mineral density were observed between groups with coated and non-coated implants and macrophage presence lightly increased in the bone grown directly in contact with the antibiotic-loaded sol-gel. In conclusion, moxifloxacin-loaded sol-gel coating is capable of preventing PJI caused by both Gram-positive and Gram-negative species.

## Introduction

Prosthetic joint infection (PJI) occurs rarely (1–2% of all cases), though its effects are often devastating due to the high associated morbidity and substantial costs of this kind of surgical complication (Tande and Patel, 2014). Additionally, the economic burden of PJI is expected to rise in the coming years with the increase in the life expectancy and number of patients undergoing arthroplasty replacements (Anagnostakos et al., 2009).

Gram-positive cocci are the most frequent microorganisms isolated from PJIs, representing up to 77% of all PJIs; of these, the most common species are *Staphylococcus aureus* and coagulase-negative staphylococci (Benito et al., 2016). However, Gram-negative bacilli can be isolated from 27–45% of all infections (Cobo et al., 2011; Benito et al., 2016) and the most commonly isolated species belong to the *Enterobacteriaceae* family, mainly *Escherichia coli*, and several non-fermenting Gram-negative bacilli. These organisms are gaining in importance given their increasing incidence and antibiotic resistance (Rodriguez-Pardo et al., 2014; Benito et al., 2016; Papadopoulos et al., 2019; Pfang et al., 2019).

Systemic and prophylactic treatment of PJIs may be ineffective, because antibiotics are unable to reach the prosthesis-tissue interface due to necrotic and/or avascular tissue that remains after surgery (Popat et al., 2007). Therefore, local antibiotic therapy was proposed as an alternative and/or adjuvant to systemic prophylaxis or treatment, preventing systemic toxicity and favoring drug release directly within the implant site (Zhao et al., 2009). Additionally, if the infection probability is reduced, the osteointegration would be improved (Gristina, 1987; Zhao et al., 2014; Martinez-Perez et al., 2017).

We recently demonstrated in an *in vitro* study that an intermediate quantity of organophosphite, [tris(trimethylsilyl) phosphite] in a sol-gel made of two silanes (3-methacryloxypropyl trimethoxysilane and 2-tetramethyl orthosilane) enhanced sol-gel adhesion on metallic surfaces and increased cell proliferation (Garcia-Casas et al., 2019).

The aim of this study was to evaluate the effect of a moxifloxacin-loaded organic-inorganic sol-gel in the *in vitro* biofilm development and treatment, cytotoxicity and cell proliferation. Finally, the efficacy of a moxifloxacin-loaded organic-inorganic sol-gel in preventing PJI caused by *S. aureus* and *E. coli* was evaluated using an *in vivo* murine model for this purpose.

## Materials and Methods

### *In vitro* Studies

#### Materials Synthesis

Titanium substrates for microbiological studies were prepared by a conventional powder metallurgy route applying a cold uniaxial charge of 7.9 tn/cm^2^ followed by a sintering step at 1,250°C for 120 min under high vacuum (10^–5^ mbar), as described elsewhere by Bolzoni et al. (2017). The starting Ti powders with a particle size below 75 μm were supplied by AP&C Inc., (Canada). The metallic substrates were ground with *SiC* paper of 1,000 grit and cleaned with ethanol in an ultrasonic bath.

**FIGURE 1:**
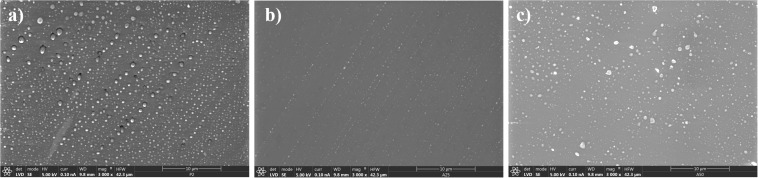
Scanning electron microscopy micrograph of different sol-gel surfaces: P2 **(a)**, A25 **(b)**, and A50 **(c)**.

Sol-gel was prepared as described elsewhere (El hadad et al., 2011) starting with a mixture of γ-methacryloxypropyltrimethoxysilane 98% (Acros Organics, Thermo Fisher Scientific, United States) and tetramethyl orthosilane 98% (Acros Organics, Thermo Fisher Scientific, United States) with a molar ratio of 1:2. Later, tris(trimethylsilyl) phosphite 92% (Sigma-Aldrich, United States) was added at a molar ratio of 1:52 with regard to the silanes (P2) (Figure 1a). Moxifloxacin (Sigma-Aldrich, United States) dissolved in water (Varanda et al., 2006) was added at two concentration: a concentration of 25 mg (A25) (Figure 1b) and a concentration of 50 mg per 20.3 mL of sol-gel (A50) (Figure 1c). These concentrations have been intentionally chosen because they represent 50% and maximum amount of this antibiotic which sol-gel can contain without compromising its stability, durability and adherence on titanium substrates.

#### Antibiotic Release From Sol-Gel

Moxifloxacin release from sol-gels coating Ti samples was evaluated by introducing in a polipropilene container containing 5 mL of phosphate bufferr saline (bioMérieux, France) previously tempered at 37°C. The antibiotic concentration was periodically measured at 1, 3, 6, 12, 24, and 48 h. The fluorimetric method previously described (Ocana et al., 2000) using an excitation/emission wavelength of 287/465 nm and a standard curve with known concentrations of antibiotic were used to estimate the antibiotic concentration at each time using 300 μL in a Polypropylene 96-well MicroWell^TM^ Plate (Thermo Fisher Scientific, United States). This experiment was performed in triplicate.

#### Microbiological Study

The biofilm formation studies were carried out using three bacterial strains: *S. epidermidis* ATCC 35984, *S. aureus* 15981 (Valle et al., 2003), and *E. coli* ATCC 25922. These species represent the most common pathogens relate to this kind of infection and serve as an example of strains susceptible to this antibiotic. All the strains were kept frozen at −80°C until experiments were performed. Overnight culture of each bacterium was grown in blood tryptic-soy agar (bioMérieux, France) at 37°C in 5% CO_2_. For each species 10^6^ colony forming units (CFU/ml) in tryptic soy broth with 1% glucose as biofilm inductive growth medium were inoculated. After incubation, the coatings were washed three times with 0.9% NaCl sterile saline (SS) (B. Braun, Germany). Adhered CFU were estimated by scraping the top disk surface with sterile wooden sticks to corroborate the viability differences on each coating. These wooden sticks with scrapped bacteria were sonicated in a 50-mL Falcon^TM^ conical tube (Thermo Fisher Scientific, United States) with 10 mL of SS, with an Ultrasons-H 3000840 low-power bath sonicator (J. P. Selecta, Spain) at 22°C for 5 min (Esteban et al., 2008). This sonicated SS was serially diluted with SS and adhered CFU were estimated using the drop plate method (Herigstad et al., 2001). Supernatant absorbance was measured at 600 nm in eight replicates in a Nunc^TM^ 96-Well Polypropylene MicroWell^TM^ Plate for estimating planktonic bacterial concentration. This experiment was performed by triplicate for each strain.

Each type of sol-gel incubated with *S. aureus* 15981 were then stained with the Live/Dead BactLight^®^ bacterial viability kit (Thermo Fisher Scientific, Waltham, MA, United States) to support visually the numerical results. Photographs (40× magnification) were taken in a DM 2000 fluorescence microscope (Leica Microsystems, Germany) for each sample. This strain was the only one which was able to be stained and photographed on sol-gels.

Mature biofilm was grown on black Nunc^TM^ F96 MicroWell^TM^ (Themo Fisher Scientific, United States) using 100 μL per well of an inoculum of 10^6^ CFU/mL of brain heart infusion (BHI) at 1% (wt./vol.) (BD, United States) plus 0.03% of fetal bovine serum (Sigma-Aldrich, United States), a culture medium that induces the formation of bacterial biofilm (Perez-Jorge et al., 2017), incubated at 37°C and 5% CO_2_ for 24 h. After incubation, the mature biofilm of each well was rinsed twice with 100 μL of SS and 200 μL of BHI at 1% (wt./vol.) with 0.03% of bovine fetal serum (Sigma-Aldrich, United States) were added. The lid of the plate was replaced by a MBECTM biofilm Incubator lid (Innovotech, Canada) whose pegs were coated twice by dipping in wells filled with 200 μL of each treatment (uncoated, P2, A25, and A50) followed by incubation at 37°C and 5% CO_2_ at 80 rpm for 48 h. Viable bacteria concentration of the grown biofilm was estimated by adding 10 μL of alamarBlue^®^ (BIO-RAD, United States) per well and was incubated at 37°C and 5% CO_2_ at 80 rpm for 1 h (Pettit et al., 2005). The fluorescence was measured using an excitation wavelength of 560 nm and an emission wavelength of 590 nm after incubation. The pegs of the uncoated material were used as positive control. This experiment was performed in eight wells per coating by triplicate for each strain.

#### Cell Study

MC3T3-E1 cells were seeded in a concentration of 10,000 cells/cm^2^ on 96-well plates with α-minimum essential medium with 10% bovine fetal serum and 1% penicillin-streptomycin (αMEM, Invitrogen, Thermo Fisher Scientific Inc., United States) and were incubated at 37°C and 5% CO_2_ overnight. After cell adherence, medium was replaced by αMEM with 50 mg/mL ascorbic acid (Sigma-Aldrich, United States), 10 mM ß-glycerol-2-phosphate (Sigma-Aldrich, United States) and different treatments: negative control, P2, A25, and A50 (*n* = 8 each) for promoting osteoblastic differentiation, and the lid of the plate was replaced by a MBECTM biofilm Incubator lid (Innovotech, Canada) whose pegs were coated by dipping in wells filled with 200 μL of each treatment (negative control, P2, A25, and A50) followed by incubation at 37°C in 5% CO_2_ for 48 h. After incubation, cytotoxicity was tested by CytoTox 96^®^ Non-Radioactive Cytotoxicity Assay (Promega, United States). Cell proliferation was determined by addition of alamarBlue^®^ solution (BIO-RAD, United States) at 10% (v/v) to the cell culture at 48 h of growth. Absorbance was measured with excitation and emission wavelengths of 570 and 600 nm, respectively, in a Tecan Infinite 200 Reader (Life Sciences, Switzerland).

### *In vivo* Model

#### Sol-Gel Synthesis and Coating of Titanium Implants

**FIGURE 2:**
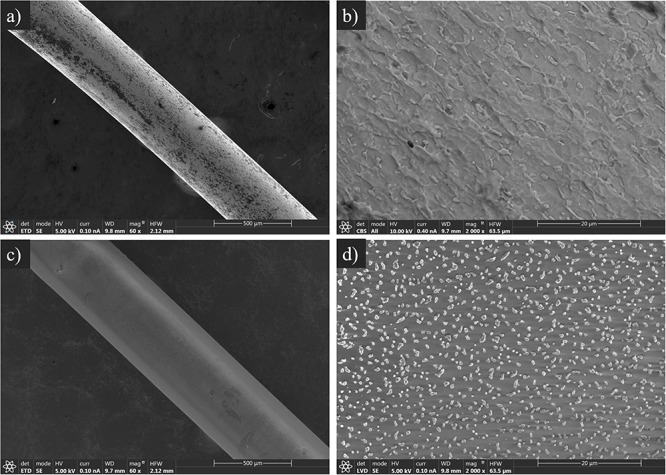
Scanning electron microscopy micrograph of CP **(a,b)** and CP+A50 surfaces **(c,d)** at 60 **(a,c)** and 2,000 **(b,d)** magnifications.

The Ti-6Al-4V implants were prepared from 0.6-mm thick Kirschner wires supplied by Depuy Synthes (Johnson & Johnson, United States). Each wire was cut into implants measuring 1 cm in length. Next, these were chemically polished (CP) as described previously (Arenas et al., 2013) to achieve a surface finish more closely resembling that used in routine clinical practice (Figures 2a,b). Finally, the Ti-6Al-4V implants for *in vivo* model were coated by dipping in A50 and allowed to dry for at least 1 h at 60°C (CP+A50) (Figures 2c,d).

#### Animal Surgical Model and Monitoring

We used two clinical strains isolated in the Clinical Microbiology department of the Fundación Jiménez Díaz University Hospital: a strain of *S. aureus* from a 62-year-old man with an acute infection of a hip prosthesis (Sa5) and a strain of *E. coli* from a 61-year-old man with another acute infection, also in a hip prosthesis (Ec30).

This study uses strains obtained from PJIs. The Instituto de Investigación Sanitaria Fundación Jiménez Díaz (IIS-FJD) Research Commission did not require the study to be reviewed or approved by an ethics committee because no clinical, demographical, analytical nor any other data from the patients were included, and bacterial strains do not need such approval according to the present legislation.

**FIGURE 3:**
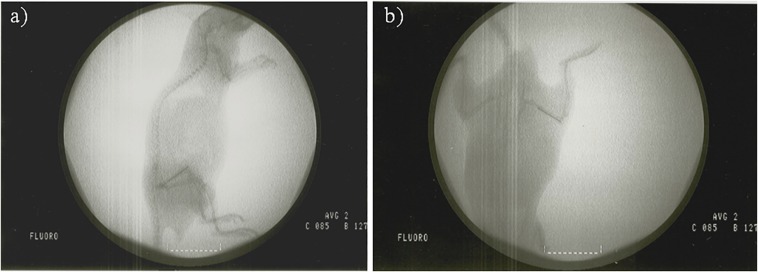
Fluoroscopy of a mouse with an implant placed in the femur in lateral **(a)** and dorsoventral **(b)** position.

Surgical intervention of the *in vivo* model was based on a modified model previously described by Lovati et al. (2013). The intervention consisted of placing the implant into the right femur of SWISS RjOrl:SWISS (CD1^®^) mice (Janvier Labs, France) through the knee using an aseptic surgical technique (Figure 3). The main modifications consisted of: (1) replacing phosphate buffer saline with saline, (2) using inhaled isoflurane (3.5%) as the only anesthesia used, and finally, and (3) using chemically polished, 1-cm-long implants made with Ti-6Al-4V instead of a 25-gauge needle, thereby simulating more realistic conditions.

Sixteen-week-old male mice with femoral implants were distributed into six groups: one group with a CP implant without infection (CP group, *n* = 8), a second group with a CP implant with infection induced by Sa5 *S. aureus* (CP Sa5 group, *n* = 5), a third group with a CP implant with infection induced by Ec30 *E. coli* (CP Ec30 group, *n* = 5), a fourth group with a CP implant coated with moxifloxacin-loaded sol-gel without infection (CP+A50 group, *n* = 8), a fifth group with a CP implant coated with moxifloxacin-loaded sol-gel with infection induced by Sa5 (CP+A50 Sa5 group, *n* = 5), and a sixth group with a CP implant coated with moxifloxacin-loaded sol-gel with infection induced by Ec30 *E. coli* (CP+A50 Ec30 group, *n* = 5).

We assessed the pain-stress and weight of each animal every 48 h on weekdays to ensure physical status. Evaluation of pain-stress consisted of the presence or absence of six behaviors directly related to pain or stress in this species and for the surgical procedure the animals had undergone: limping, piloerection, lack of grooming, wound presence, passivity, and aggressiveness. In cases of sustained weight loss over time, the most appropriate refinement measures were taken to encourage the animal to eat. For this, they were offered an additional mixture of grains and vegetables (Vitakraft, Bremen, Deutschland). At 15 days postsurgically, a urine sample was taken from all infected animals, and bacteria presence was evaluated qualitatively.

Five weeks after surgery, the animals were euthanized using hypercapnia. The entire femur of each animal was then recovered through sterile preparation of the knee, including surgical field isolation.

#### Microbiological Study

The biofilm formation ability of the different clinical strains was evaluated according to a widely accepted published protocol (Stepanovic et al., 2007). The strains were transferred from stock culture into blood tryptic-soy agar and incubated at 37°C overnight under aerobic conditions. The next day, colonies were suspended in TSB until a turbidity comparable to 0.5 MacFarland scale (∼10^8^ CFU/mL) was reached. This suspension was diluted 1:100 in TSB+1% glucose to reach a bacterial concentration of approximately 10^6^ CFU/mL. Then, 200 μL from the diluted suspension was aliquoted into 96-well untreated microtiter plate and incubated at 37°C and 5% CO_2_ for 24 h under static aerobic conditions. The next day, the wells were aspirated, and each well was washed three times with 200 μL SS. After washing, the remaining attached bacteria were fixed with 200 μL of methanol, and incubated at room temperature for 20 min. The methanol was discarded and plates were left to dry in at 60°C. Finally, the biofilm formed was stained with 200 μL of 2% crystal violet for Sa5 and of 1% safranin for Ec30 for 15 min. Plates were washed two times with 250 μL of sterile distilled water, and dye bound to the cells was eluted with 250 μL of absolute ethanol. The absorbance was measured at 570 nm for crystal violet and at 492 nm for safranin using a microplate reader. Experiments were performed in eight wells and repeated three times. The cut-off value (optical density control, ODC) was defined as three standard deviations above the mean OD of the negative control. The strains were classified according to its optical density (OD) per well within of the following categories: non-biofilm former (OD ≤ ODC), weak biofilm former (ODC < OD < 2 × ODC), moderate biofilm former (2 × ODC < OD < 4 × ODC) and strong biofilm former (OD > 4 × ODC).

The urine samples were seeded in CHROMID^®^ CPS^®^ Elite (bioMérieux, France) to diagnose possible bacteriuria.

Using a hammer, each femur was divided into two samples on a sterile surface: (1) bone and adnexa, and (2) implant.

The bone was immersed in 2 mL of SS and sonicated using an sonicator at 22°C for 5 min (Esteban et al., 2008). The resulting sonicate was diluted in a 10-fold dilution bank and seeded on blood-chocolate agar (bioMérieux, France) using the plaque extension method, which consists of seeding 100 μL/plate of each dilution. The concentration of bacteria was estimated as CFU/g of bone and adnexa.

The implant was sonicated in 2 mL of saline for 5 min to release the adhered biofilm bacteria and estimated their concentration, measured as CFU/cm^2^ of implant. All plates were checked at 24 and 48 h.

#### Microcomputed Tomography

Three bone samples from each non-infected control group included in the aforementioned model were fixed in 10% formaldehyde for 48 h at 4°C. After fixation, they were dehydrated in 96% ethanol for 48 h, changing the ethanol every 24 h, and in 100% ethanol for 48 h, changing the ethanol every 24 h.

Three-dimensional microcomputed tomography was performed with a CompaCT scanner (Sedecal, Spain). Data were acquired with 720 projections by 360-degree scan, having an integration time of 100 ms with three frames, photon energy of 50 KeV, and a current of 100 μA. The duration of imaging was 20 min per scan. Three-dimensional renderings of the hind paws were generated using original volumetric images reconstructed with MicroView software (GE Healthcare). Bone mass (BM, mg), bone volume (BM, cm^3^), and bone mineral density (BMD = BM/BV, mg/cm^3^) were quantified from MicroCT scans using GE MicroView software 2.2.

#### Histological Studies

Three femurs of CP group and CP+A50 group were fixed in 4% paraformaldehyde for 48 h, decalcified in 10% EDTA for 4 weeks, paraffin-infiltrated, and stained with hematoxylin-eosin. Implants were removed and transversal sections in the knee condyles (5 μm) were done.

Tartrate-resistant acid phosphatase (TRAP) staining was carried out in paraffin sections with a homemade TRAP buffer (0.1 M acetate buffer, 0.3 M Sodium Tartrate 10 mg/ml Naphtol AS-MX phosphate, 0.1% Triton X-100, 0.3 mg/ml Fast Red Violet LB (Sigma-Aldrich, United States). After deparaffinization and acetate buffer washing processes, samples were incubated in TRAP buffer for 30 min and counterstained with Fast Green.

Immunohistochemistry analysis was carried out as previously described (Mediero et al., 2012). Briefly, sections were incubated with proteinase K Solution (20 μg/mL in TE Buffer, pH 8.0) for 15 min in water bath at 37°C for antigen retrieval after deparaffinization and re-hydratation. Blocking of non-specific binding was performed with PBS 3% BSA and 0.1% Triton X-100 for 1 h, and primary antibodies anti-cathepsin K (1:25), CD68 (1:200) and alkaline phosphatase (ALP) 1:200 (all antibodies from Santa Cruz Biochenology, United States) were incubated overnight at 4°C in a humidifying chamber. Secondary antibodies goat anti-rabbit-FITC (1:200), goat anti-mouse-FITC (1:200) (Invitrogen, Life Technologies, United States) were incubated for 1 h in the dark. Slides were mounted with Fluoroshield with DAPI mounting media (Sigma-Aldrich, United States). Images were taken with the iScan Coreo Au scanner (Ventana Medical Systems, Roche diagnostics, Spain) and visualized with Image Viewer v.3.1 software (Ventana Medical Systems, Roche diagnostics, Spain). Images were taken at 4× or 10× magnifications.

### Statistical Analysis

Statistical analyses were performed using Stata Statistical Software, Release 11 (StataCorp, United States). Data were evaluated using a one-sided Wilcoxon non-parametric test to compare two groups. Statistical significance was set at *p*-values ≤ 0.05. Body weight was evaluated over time using a linear regression model. Microbiological, cellular results and weight values are represented as median and interquartile range. Other behavioral variables are represented as relative frequencies at each time point.

## Results

### *In vitro* Studies

#### Antibiotic Release From Sol-Gel

**FIGURE 4:**
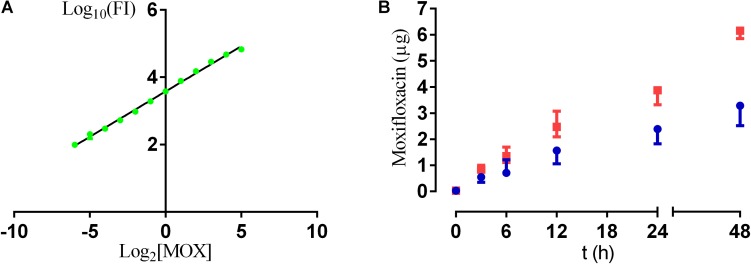
Calibration curve resulting from known concentrations of moxifloxacin (MOX) and their fluorescence intensity (FI) **(A)**; and amount of moxifloxacin released from A25 (blue) and A50 (red) over time **(B)**.

Figure 4A represents the standard curve with known concentrations of antibiotic and their repectives fluorescence intensity. The regression coefficients (*R*^2^) was 0.9918. The antibiotic release shows a lineal release over time both from A25 and from A50 (Figure 4B), and maximum release values were obtained at 48 h for both sol-gels. The moxifloxacin release rate from A50 (120 ng/h) was significantly higher than the rate from A25 (60.6 ng/h) (*p*-value < 0.0001 for *t*-Student test).

#### Microbiological Studies

**FIGURE 5:**
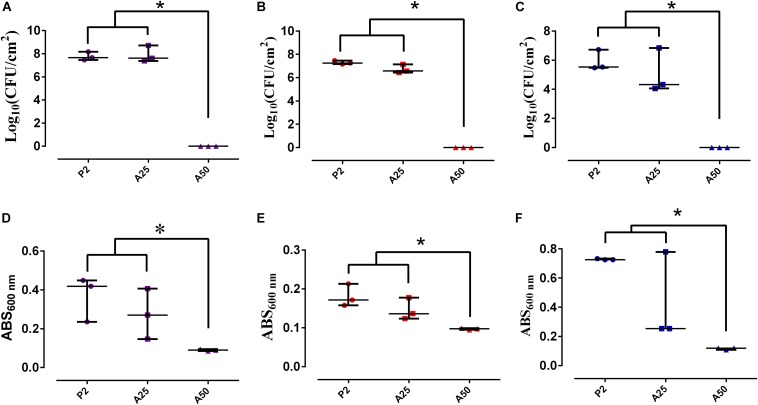
Bacterial biofilm concentration per area unit (CFU/cm^2^) and planktonic bacterial concentration (ABS) of *S. epidermidis* ATCC 35984 **(A,D)**, *S. aureus* 15981 **(B,E)**, and *E. coli* ATCC 25922 **(C,F)** on different types of coatings: P2, A25 and A50. ^∗^*p*-value < 0.05 for Wilcoxon test.

**FIGURE 6:**
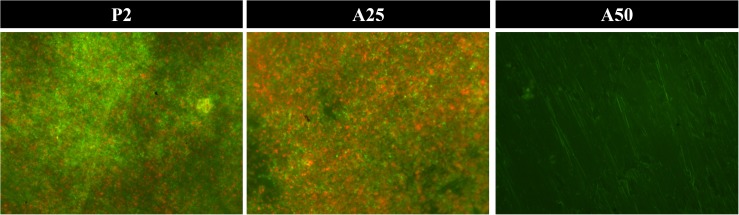
*Staphylococcus aureus* biofilm grown on each sol-gel and stained using Live/Dead Bactlight to 40× magnification. Green bacteria are viable and red ones are dead.

Figures 5A–C shows plots of the bacteria concentration per area unit that are attached to the surface by means of colony forming units (CFU). CFU per square centimetre significantly decreased only on A50 for the three strains used (*p*-value < 0.05). Plots of Figures 5D–F show that the planktonic bacterial concentration is proportional to the absorbance of the supernatant measured at 600 nm. Bacterial absorbance was inversely proportional to moxifloxacin concentration. The microbiological study revealed two responses for the biofilm development and for the planktonic bacterial concentration. The biofilm development response was all-or-nothing for the staphylococci when their biofilms grow in presence of P2 or A25 (all) and A50 (nothing) (Figures 5A,B, 6). *E. coli* ATCC 25922 biofilm development showed a lower development which was influenced by the concentration of antibiotic (Figure 5C). On the contrast, the planktonic bacterial concentration was gradually and inversely proportional to the moxifloxacin coating concentration for all the tested strains (Figures 5D–F).

**FIGURE 7:**
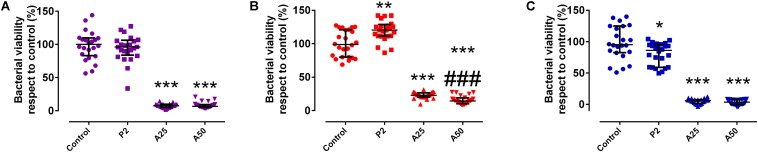
Percentage of biofilm growth respect to the control of *S. epidermidis* ATCC 35984 **(A)**, *S. aureus* 15981 **(B)**, and *E. coli* ATCC 25922 **(C)** on different types of coatings: P2, A25 and A50. ^∗^*p*-value < 0.05, ^∗∗^*p*-value < 0.01, ^∗∗∗^*p*-value < 0.001 for Wilcoxon test between biofilm growth on the control and on coatings loaded with MOX. ^###^*p*-value < 0.001 for Wilcoxon test between biofilm growth on A25 and A50.

Coatings containing antibiotic behaved as inhibitors of the growth of a mature biofilm (Figure 7). The unloaded coating (P2) did not show an inhibitor behavior in the presence of staphylococci mature biofilms (Figures 7A,B) (*p*-value > 0.05) but showed, however, a slightly inhibitor behavior in the presence of a mature biofilm *E. coli* (*p*-value = 0.0088) (Figure 7C). Though A25 and A50 are able to inhibit in a similar way the mature biofilm growth of *S. epidermidis* ATCC 35984 and *E. coli* ATCC 25922, A50 showed the highest inhibitor behavior on the mature biofilm of *S. aureus* 15981 compared to A25 (*p*-value < 0.001).

#### Cellular Studies

**FIGURE 8:**
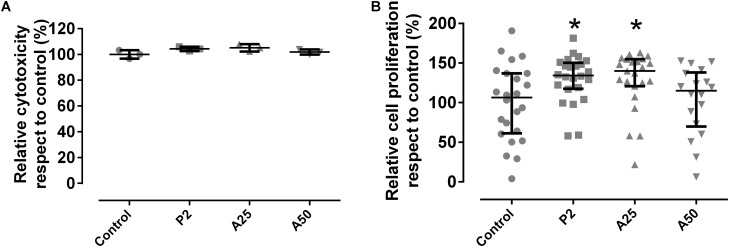
Cytotoxicity **(A)** and cell proliferation **(B)** of MC3T3-E1 cells when growing in presence of each coating. Control was considered in the presence of any coating. ^∗^*p*-value < 0.05 for Wilcoxon test between cell proliferation on control and on different coatings loaded with MOX.

Cytotoxicity was not detected in the presence of the coatings tested respect to the control used (*p*-value > 0.05) (Figure 8A).

This explains why P2 stimulates significantly up to 30% the cell proliferation compared to the other coatings and the control (Figure 8B). However, cell proliferation was also influenced by the antibiotic added to the coatings. A50 showed a lower percentage of cell proliferation than the control, meaning the high quantity of antibiotic added on A50 acted against cells although only in the first 48 h.

### *In vivo* Studies

#### Animal Monitoring

**FIGURE 9:**
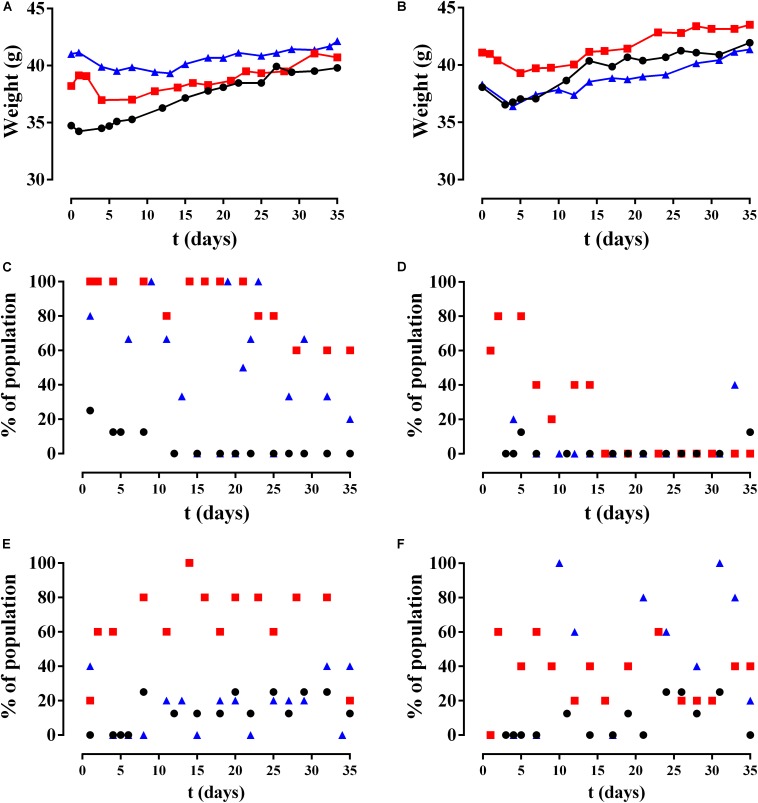
Results of animal monitoring over time. **(A,B)** Mean weight, **(C,D)** limping, and **(E,F)** piloerection in different non-infected groups (circles), Sa5-infected group (squares), and Ec30-infected group (triangles) with CP (left column) and CP+A50 (right column).

The mean weight over time of the mice is shown by group in Figures 9A,B. All groups significantly increased their weight over time (*p*-value < 0.001) except CP Ec30 (*p*-value = 0.1196).

Only two of the six behavioral variables evaluated showed a modification during the study: limping and piloerection (Figures 9C,D). Limping was significantly more frequent in the CP Sa5 group than the CP+A50 Sa5 groups (*p*-value < 0.0001) and was significantly higher in the CP Ec30 group compared to the CP+A50 Ec30 group (*p*-value < 0.0001) (Figure 9C). Piloerection was observed with significantly greater frequency in the CP Sa5 group than in the CP+A50 Sa5 groups (*p*-value = 0.0004) and increased significantly over time in the CP+A50 Ec30 group compared to the CP Ec30 group (*p*-value = 0.0073) (Figures 9E,F).

#### Microbiological Study

Both of the clinical bacterial strains used were strong biofilm former: 12.2 (9.3–15.5)×ODC for Sa5 and 4.3 (4.2–4.8)×ODC for Ec30.

Bacteriuria was only detected in nine of the Ec30-infected mice: four from the CP Ec30 group and five from the CP+A50 Ec30 group. The antibiotype of *E. coli* strains isolated from urine was identical to that of Ec30 strains inoculated during surgery.

**FIGURE 10:**
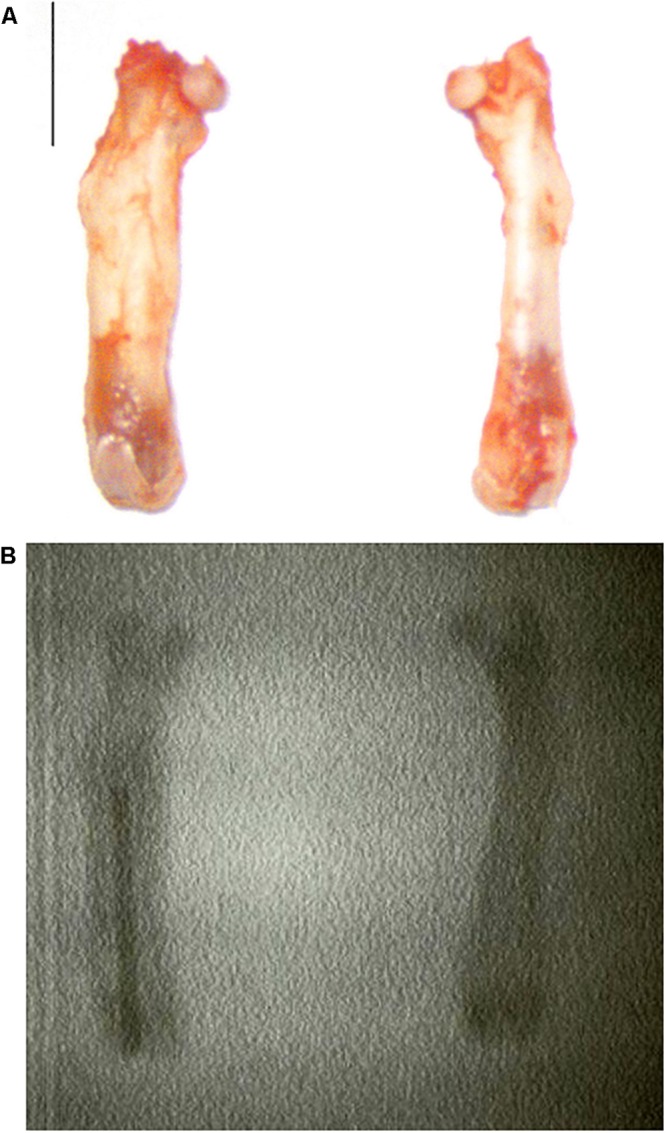
Photograph of a femur without (**A**, right) and with osteomyelitis (**A**, left) and their radiographic image **(B)**. The bar represents 0.5 cm.

Of all the femurs studied, seven showed macroscopic deformation consistent with osteomyelitis (Figure 10): five animals in the CP Sa5 group and two in the CP Ec30 group. Deformation was higher in CP Sa5 than in CP Ec30.

**FIGURE 11:**
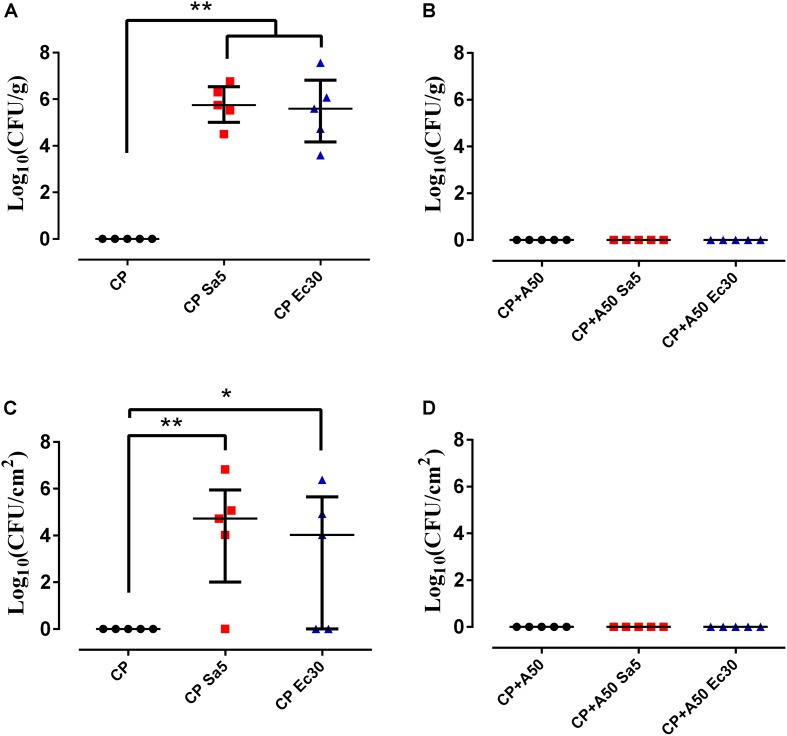
Quantity of bacteria in bone and adnexa (left column) and on recovered implant (right column) with CP **(A,B)** and CP+A50 **(C,D)**. ^∗^*p*-value < 0.05, ^∗∗^*p*-value < 0.01 for Wilcoxon test between non-infected group and infected groups by *S. aureus* (Sa5) or *E. coli* (Ec30).

Statistically significant differences were observed between the quantity of bacteria in bone and adnexa (*p*-value = 0.0027 for CP and CP Sa5, and *p* = 0.0027 for CP and CP Ec30) and implants (*p*-value = 0.0093 for CP and CP Sa5, and *p* = 0.0275 for CP and CP Ec30) between the CP groups (Figures 11A,B).

No differences were observed in the number of bacteria in bone and adnexa and implants (*p*-value = 1.0000 for CP+A50 and CP+A50 Sa5, and *p* = 1.000 for CP+A50 and CP+A50 Ec30) between the CP+A50 groups (Figures 11C,D).

#### Microcomputed Tomography

**FIGURE 12:**
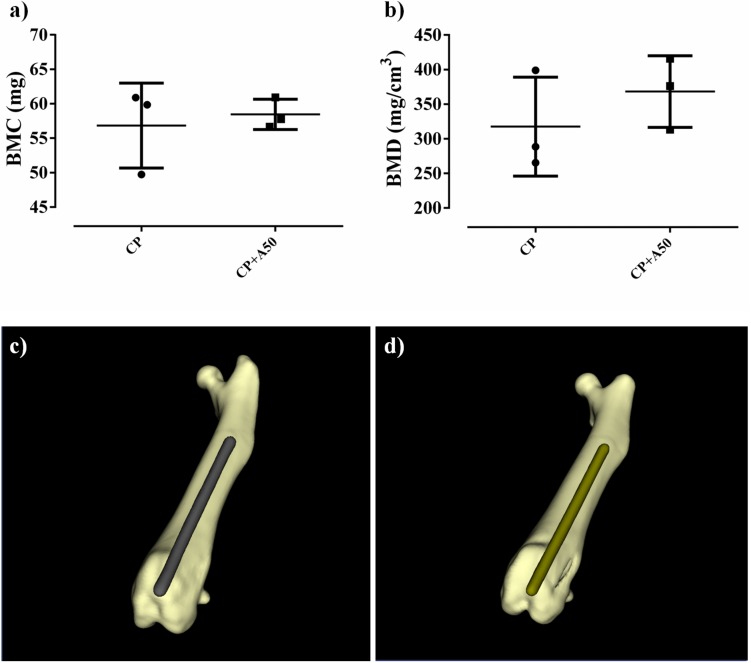
Bone mineral content (BMC) **(a)** and bone mineral density (BMD) **(b)** and their three-dimensional reconstructions of a representative sample of the CP group **(c)** and CP+A50 group **(d)**.

No differences were observed between the BMC and BMD of non-infected CP and non-infected CP+A50 (*p*-value = 0.1376 and *p* = 0.4137, respectively) (Figure 12). The BMD results were perfectly comparable, since BMC was no different between the groups compared.

#### Histology

**FIGURE 13:**
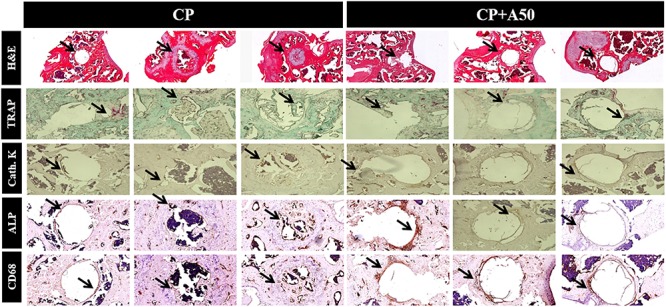
Immunohistochemistry for markers of different bone cells. Long bones were processed and immunohistologic staining carried out. Shown are representative images stained for haematoxilin-eosin (H&E), tartrate-resistant acid phosphatase (TRAP) staining, cathepsin K (cath. K), alkaline phosphatase (ALP) and macrophages (CD68). H&E images were taken at 4× magnification. All immunostaining images were taken at 10× magnification.

Hematoxylin-eosin staining showed that mice with CP implant presented fibrous tissue in the area where implant was present (arrow) (Figure 13). CP+A50 showed absence of tissue what correlated with the area occupied by the implant (arrow) (Figure 13). When bone markers were studied, no many changes were found among different animals. Both groups showed similar number of TRAP positive cells (arrow), and immunostaining for cathepsin K (cysteine protease, osteoclast marker) did not showed any difference, ALP (alkaline phosphatase) positive cells were similar in CP and CP+A50, but macrophages (CD68 + cells) were increased in CP+A50.

## Discussion

In this study, we report the efficacy of a novel approach of sol-gel coatings containing moxifloxacin, that was demonstrated to provide an anti-infective surface to Ti-based materials both *in vitro* and *in vivo*. The active surfaces prevented locally the biofilm development and treated mature biofilms of different clinically important bacterial species. Finally, we demonstrated the *in vivo* efficacy of moxifloxacin-loaded sol-gel in preventing PJI caused by Gram-positive *S. aureus* and Gram-negative *E. coli*.

The microbiological study revealed that only A50 completely inhibited the formation of biofilm on the coated surface and was the best treatment for a mature biofilm of the three bacterial species. Hence, A50 was the most appropriate coating to use in the *in vivo* model and would be the best prophylactic treatment in the clinical practise. The moxifloxacin release rate of A50 was almost two-fold higher than A25 and showed a lineal trend over time as other antibiotics (e.g., vancomycin) loaded in sol-gels made of tetraethyl orthosilicate (Radin and Ducheyne, 2007).

Commonly reported symptoms of PJI include pain, joint swelling or effusion, erythema or warmth around the joint, fever, drainage, or the presence of a sinus tract communicating with the arthroplasty (Tande and Patel, 2014). The joint pain in our *in vivo* model was monitored by observing limping, and generalized malaise was monitored by observing evidence of piloerection often associated with infection (Campos et al., 2016). Infected animals with A50-coated implants showed significantly less limping and piloerection over time than infected animals with non-coated implants, with the exception of piloerection in *E. coli*-infected animals with coated implants (Figure 9). In these cases of *E. coli*-infected animals with coated implants, piloerection was higher in those animals with coated implant compared to animals with non-coated implants; this may be due to the bacteriuria observed in these animals. We cannot completely explain this apparently symptomatic bacteriuria, though it may be caused by bacterial concentration in the bladder due to transient bacteraemia (Wang et al., 2017) during surgery as well as the motility of *E. coli* (Kaya and Koser, 2012), which allows the bacteria to migrate and colonize a new niche with lower hostility than the interior of the bone when it contains an antibiotic-coated implant. The microbiological results from the *in vivo* study revealed that moxifloxacin-loaded sol-gel showed an all-or-nothing response when used to coat the implant and *S. aureus* or *E. coli* infection is induced (Figure 11) as expected. The femoral deformation observed in *S. aureus*-infected groups with non-coated implants is consistent with the ability of *S. aureus* to internalize in osteoblasts (Cremet et al., 2015) and cause deforming osteomyelitis in chronic bone infections (de Mesy Bentley et al., 2017) (Figure 10). In the case of *E. coli*, the bone deformation observed in infected groups with non-coated implants may be due to osteoblastic cytotoxicity (Cremet et al., 2015).

The non-cytotoxicity of these coatings was validated and the cellular proliferation was slightly favored in presence of P2 and A25. The addition of tris(trimethylsilyl) phosphite to the sol-gel formulation created localized areas with higher phosphorus content that is released in form of phosphate when sol-gel is degraded over time. This inorganic phosphorus compound significantly stimulates the growth and osteogenic differentiation of the bone cells (Liu et al., 2009). This cell proliferation increase was absent in presence of A50, this may be due to that the proliferative effect exerted by the phosphate released from sol-gel degradation would be counteracted by the high moxifloxacin concentration, an fluoroquinolone antibiotic which has been frequently associated with a wide spectrum of musculoskeletal complications that involve not only tendon but also cartilage, bone, and muscle (Hall et al., 2011; Jacobs et al., 2014). Due to the degradation of sol-gel would take place after 48–72 h of surgery, this potential local cytotoxicity would be a assumable and transient complication by taking into account the beneficit of prophylaxis. Moreover, moxifloxacin-loaded coating showed a non-harmful effect on bone mineralization according to the microcomputed tomographic images (Figure 12) and no changes in bone markers were found among groups (Figure 13), what correlates with micro-CT data. The increased in macrophages in CP+A50 group might be indicating that the local antibiotic delivery from sol-gel is recruiting macrophages at site of surgery what would impede the bacterial proliferation, since it has been also found previously that a multifunctional nanogel for targeted vancomycin delivery provides macrophage targeting and lesion site−activatable drug release properties, which enhances bacterial growth inhibition in a zebra fish *in vivo* model (Xiong et al., 2012).

In recent years, different types of coating have been introduced for clinical use: natural, peptide, ceramic, and synthetic coatings (Civantos et al., 2017). Most have been designed with osteointegration (Civantos et al., 2017; Haimov et al., 2017) and antibacterial purposes (Zhao et al., 2009; Swartjes et al., 2015; Kulkarni Aranya et al., 2017). To date, antibacterial, biodegradable perioperative coatings are designed to be loaded with gentamicin or vancomycin (Romanò et al., 2015) and are already on the market. One example is a fast-resorbable, antibiotic-loadable hydrogel composed of covalently linked hyaluronan and poly-D,L-lactide, whose efficacy has been proven *in vitro* (Drago et al., 2014) and in a multicentre randomized controlled trial (Malizos et al., 2017). Thus, the moxifloxacin-loaded sol-gel used in our study is a new, cost-effective alternative for locally preventing and without significant compromising the bone mineralization. Nevertheless, moxifloxacin can be replaced by a broad spectrum of antibiotics according to the clinical need. The use of this biomaterial and its versatility represent an important advance in Traumatology and Orthopedics field.

In conclusion, coating loaded with higher concentration of moxifloxacin (A50) showed excellent bactericidal and broad-spectrum anti-biofilm response since it showed the highest inhibitor behavior on the biofilm development (local prevention) and on mature bacterial biofilm (local treatment).

## Data Availability Statement

The datasets generated for this study are available on request to the corresponding author.

## Ethics Statement

This study was approved by the Instituto de Investigación Sanitaria Fundación Jiménez Díaz (IIS-FJD) Animal Care and Use Committee, which includes *ad hoc* members for ethical issues. Animal care and maintenance complied with institutional guidelines as defined in national and international laws and policies (Spanish Royal Decree 53/2013, authorization reference PROEX019/18 March 8, 2018 granted by the Counsel for the Environment, Local Administration and Territorial Planning of the Community of Madrid and, Directive 2010/63/EU of the European Parliament and of the Council of September 22, 2010).

## Author Contributions

JA-C, AG-C, AM, AJ-M, and JE made substantial contributions to the research design, acquisition, analysis, interpretation of the data, drafting the manuscript and revising it critically, and approved the submitted and final versions of the manuscript. DR, IC-L, and FM made substantial contributions to the research design, acquisition, analysis and interpretation of data and approved the submitted and final versions of the manuscript.

## Conflict of Interest

JE received travel grants from Pfizer and conference fees from bioMérieux and Heraeus. The remaining authors declare that the research was conducted in the absence of any commercial or financial relationships that could be construed as a potential conflict of interest.
